# Early prediction of neoadjuvant chemotherapy response for advanced breast cancer using PET/MRI image deep learning

**DOI:** 10.1038/s41598-020-77875-5

**Published:** 2020-12-03

**Authors:** Joon Ho Choi, Hyun-Ah Kim, Wook Kim, Ilhan Lim, Inki Lee, Byung Hyun Byun, Woo Chul Noh, Min-Ki Seong, Seung-Sook Lee, Byung Il Kim, Chang Woon Choi, Sang Moo Lim, Sang-Keun Woo

**Affiliations:** 1 Department of Nuclear Medicine, Asan Medical Center, University of Ulsan College of Medicine, Seoul, Republic of Korea; 2 Department of Nuclear Medicine, Korea Cancer Center Hospital, Korea Institute of Radiological and Medical Sciences (KIRAMS), Seoul, Republic of Korea; 3 Division of RI-Convergence Research, Korea Institute of Radiological and Medical Sciences (KIRAMS), Seoul, Republic of Korea; 4 Department of Surgery, Korea Cancer Center Hospital, Korea Institute of Radiological and Medical Sciences (KIRAMS), Seoul, Republic of Korea; 5 Department of Pathology, Korea Cancer Center Hospital, Korea Institute of Radiological and Medical Sciences (KIRAMS), Seoul, Republic of Korea

**Keywords:** Diagnostic markers, Molecular imaging

## Abstract

This study aimed to investigate the predictive efficacy of positron emission tomography/computed tomography (PET/CT) and magnetic resonance imaging (MRI) for the pathological response of advanced breast cancer to neoadjuvant chemotherapy (NAC). The breast PET/MRI image deep learning model was introduced and compared with the conventional methods. PET/CT and MRI parameters were evaluated before and after the first NAC cycle in patients with advanced breast cancer [n = 56; all women; median age, 49 (range 26–66) years]. The maximum standardized uptake value (SUVmax), metabolic tumor volume (MTV), and total lesion glycolysis (TLG) were obtained with the corresponding baseline values (SUV0, MTV0, and TLG0, respectively) and interim PET images (SUV1, MTV1, and TLG1, respectively). Mean apparent diffusion coefficients were obtained from baseline and interim diffusion MR images (ADC0 and ADC1, respectively). The differences between the baseline and interim parameters were measured (ΔSUV, ΔMTV, ΔTLG, and ΔADC). Subgroup analysis was performed for the HER2-negative and triple-negative groups. Datasets for convolutional neural network (CNN), assigned as training (80%) and test datasets (20%), were cropped from the baseline (PET0, MRI0) and interim (PET1, MRI1) images. Histopathologic responses were assessed using the Miller and Payne system, after three cycles of chemotherapy. Receiver operating characteristic curve analysis was used to assess the performance of the differentiating responders and non-responders. There were six responders (11%) and 50 non-responders (89%). The area under the curve (AUC) was the highest for ΔSUV at 0.805 (95% CI 0.677–0.899). The AUC was the highest for ΔSUV at 0.879 (95% CI 0.722–0.965) for the HER2-negative subtype. AUC improved following CNN application (SUV0:PET0 = 0.652:0.886, SUV1:PET1 = 0.687:0.980, and ADC1:MRI1 = 0.537:0.701), except for ADC0 (ADC0:MRI0 = 0.703:0.602). PET/MRI image deep learning model can predict pathological responses to NAC in patients with advanced breast cancer.

## Introduction

Neoadjuvant chemotherapy (NAC) has been established as the standard treatment for advanced breast cancer^1^. Pathological examination is essential after breast surgery for evaluating the response to NAC^2^. Furthermore, a complete pathological response to NAC is considered to be a critical prognostic factor for favorable outcomes^3,4^. Early identification of non-responders is clinically valuable because these patients need aggressive treatment. Moreover, the use of ineffective, toxic chemotherapy should be avoided in responders.

Various conventional imaging modalities have been used to evaluate the response to NAC before surgery, including fluorodeoxyglucose positron emission tomography/computed tomography (FDG-PET/CT) and magnetic resonance imaging (MRI). FDG-PET/CT studies have shown that decreased tumor metabolism can differentiate responders from poor responders to NAC. Dynamic contrast-enhanced MRI has been shown to predict histopathological responses based on changes in tumor size and transfer constant^5,6^. However, the differences in outcomes and relatively small sample sizes have rendered a comparison of these FDG-PET/CT and MRI studies inconclusive.

Deep learning is an emerging technique for solving problems that have persisted in the artificial intelligence community. Contrary to traditional machine learning methods including linear regression, logistic regression, the Naïve Bayes classifier, and support vector machines (SVMs), deep learning algorithms recruit multiple, deep layers of perceptions that capture both low- and high-level representations of data^7,8^. Convolutional neural networks (CNNs) are a subclass of deep neural networks that employ a specialized mathematical function, known as a “convolution”^9^. The basic concept of CNNs originated from the biological mechanisms of visual recognition in the feline primary visual cortex^10^. The CNN algorithm based AlexNet was proposed by Krizhevsky et al. in 2012^11^. Its effective performance, compared to that of traditional machine learning (e.g., logistic regression [LR]) methods, garnered attention for image recognition tasks. Since then, several models based on deep learning techniques have been developed for image recognition. Application of the deep learning method of CNNs to medical images has been subjected to increased attention^12,13^. Moreover, deep learning methods are widely used for the diagnosis and detection of breast cancer with mammography and MRI^14–16^. CNNs are widely used for classification purposes. CNN-based software includes U-Net that was designed for biomedical image segmentation and V-Net that was designed for volumetric medical image segmentation^17–19^.

However, there are no published studies on the use of PET/CT and MRI for predicting the responses of breast cancer treatment, with the help of deep learning methods. The primary aim of this study was to investigate the application of CNNs in predicting patient responses to NAC for advanced breast cancer using PET and MRI. The secondary aim was to compare the predictive values obtained from CNNs with that of conventional imaging parameters.

## Materials and methods

### Patient enrollment

We retrospectively reviewed the prospective study data of 119 patients who visited Korea Cancer Center Hospital from August 2009 to February 2016. The inclusion criteria were as follows: (1) age 17 years or above, (2) the participant had to be a woman, (3) histopathologically proven American Joint Committee on Cancer (AJCC) stage II or III breast cancer, and (4) patients who underwent PET/CT and MRI before and 3 weeks after the first cycle of NAC. The exclusion criterion was a tumor size of less than 2 cm based on the imaging findings. Sixty-three patients were excluded. Thus, 56 patients were selected. The study was approved by the Institutional Review Board of KIRAMS (IRB No.: KIRAMS 2019-01-003), which waived the requirement for informed consent. All methods were performed in accordance with the relevant guidelines and regulations.

All patients received three cycles of doxorubicin (50 mg/m^2^) combined with docetaxel (75 mg/m^2^) once every 3 weeks as NAC. Mastectomy or breast-conserving surgery with axillary lymph node dissection was performed after 2 weeks. All patients received another three cycles of chemotherapy postoperatively. Patients with hormone receptor-positive breast cancer received additional hormone therapy. Patients positive for human epidermal growth factor receptor-2 (HER2) also received trastuzumab therapy for 1 year after surgery.

### FDG-PET/CT and MRI

Each patient underwent a sequential whole-body PET/CT scan (Biograph 6; Siemens Medical Solutions, Malvern, PA, USA) and a 3.0-T whole-body MRI scan (MAGNETOM Trio A Tim; Siemens Medical Solutions, Erlangen, Germany) concurrently. Patients fasted for at least 6 h before intravenous administration of 18F-FDG (7.4 MBq/kg). The blood glucose levels of all patients were checked to ensure it was below 7.2 mmol/L at this time. The patients were made to lie down in a silent room under stable conditions for 60 min, following intravenous infusion of 18F-fluorodeoxyglucose (FDG). FDG-PET/CT was performed 60 min after FDG injection, followed by MRI 90 min after the FDG injection. PET images were reconstructed using CT data for attenuation correction using the 2D ordered-subsets expectation maximization (2D OSEM) algorithm. PET parameters were as follows: field of view, 700 mm; matrix size, 256 × 256; Full width at half maximum (FWHM), 4.0 mm.

MR images of both breasts were acquired using a 3.0-T whole-body MRI scanner with a dedicated phased-array breast coil, while the patients in the prone position. We used the following parameters: TR/TE, 6100/78 ms; matrix size, 100 × 128; field of view, 380 mm; receiver bandwidth, 3004 Hz/pixel; slice thickness, 4 mm; acquisition time, 4 min 22 s; voxel size, 0.9 × 0.6 × 3.0 mm. Diffusion-weighted images were acquired using a spin-echo type single-shot echo-planar imaging sequence. Imaging for apparent diffusion coefficient (ADC) was performed with b values of 0 and 800 s/mm^2^. The parameters used in diffusion-weighted images were as follows: field of view, 420 mm; slice thickness, 4 mm; TR/TE, 6600/86 ms; voxel size, 2.2 × 2.2 × 4.0 mm. Diffusion images were obtained in the three orthogonal directions to calculate the ADC maps. Dynamic MR images were integrated using a three-dimensional fat-suppressed volumetric interpolated breath-hold examination (VIBE) sequence before contrast agent administration and five dynamic series at 78, 144, 210, 300 and 366 s after contrast agent administration using the following parameters: TR/TE 3.95/1.49 ms; flip angle 10°; field of view 340 mm; slice thickness 1 mm; matrix size 318 × 448; acquisition time 7 min 19 s. All patients were injected a bolus of 0.1 mmol/kg Gd-DTPA-BMA (gadodiamide, Omniscan; GE Healthcare) intravenously at a rate of 1.5 mL/s using a power injector, followed by a flush with 20 mL saline. FDG PET/CT and MR images were co-registered using the syngo FusedVision 3D software (Siemens Medical Solutions, Erlangen, Germany).

### Image analysis

We drew an ellipsoid volume of interest including the entire primary tumor, and measured the maximum standardized uptake value (SUVmax). The largest cross-sectional area was used for multiple lesions. Metabolic tumor volume (MTV) was calculated automatically by adding the volume of voxels to the threshold SUV value of 2.5. Total lesion glycolysis (TLG) was calculated by multiplying MTV and mean SUV with the threshold SUV value of 2.5. The ADC value was obtained from the diffusion MRI dataset. We carefully placed a circle-shaped ROI inside the tumor on the ADC map that best coincided with the largest well-contrast cross-sectional area of the T1 image, side by side. The mean ADC value with ROI was recorded. Tumor size was estimated with each MRI examination as the product of the largest diameter on the enhancing tumor. Other variables of dynamic contrast images were not adopted in this study due to multiparmetric variables and different time points.

According to conventional imaging parameters, SUV0, MTV0, and TLG0 were determined from the SUV, MTV, and TLG of PET values obtained at baseline. SUV1, MTV1, and TLG1 were obtained in a similar manner to the interim images, which were obtained 3 weeks after the first cycle of NAC. ADCmean of the ADC images obtained at baseline was defined as ADC0. ADCmean of the interim images was defined as ADC1. The following parameters were calculated to assess the differences between the baseline and interim images:

$$ \Delta {\text{SUV }}\left( \% \right) \, = \, \left( {{\text{SUV1}}{-}{\text{SUV}}0} \right) \times {1}00/{\text{SUV}}0 $$

$$ \Delta {\text{MTV }}\left( \% \right) \, = \, \left( {{\text{MTV1}}{-}{\text{MTV}}0} \right) \times {1}00/{\text{MTV}}0 $$

$$ \Delta {\text{TLG }}\left( \% \right) \, = \, \left( {{\text{TLG1}}{-}{\text{TLG}}0} \right) \times {1}00/{\text{TLG}}0 $$

$$ \Delta {\text{ADC }}\left( \% \right) = \, \left( {{\text{ADC1}}{-}{\text{ADC}}0} \right) \times {1}00/{\text{ADC}}0 $$

### Deep learning technique

**Figure 1:**
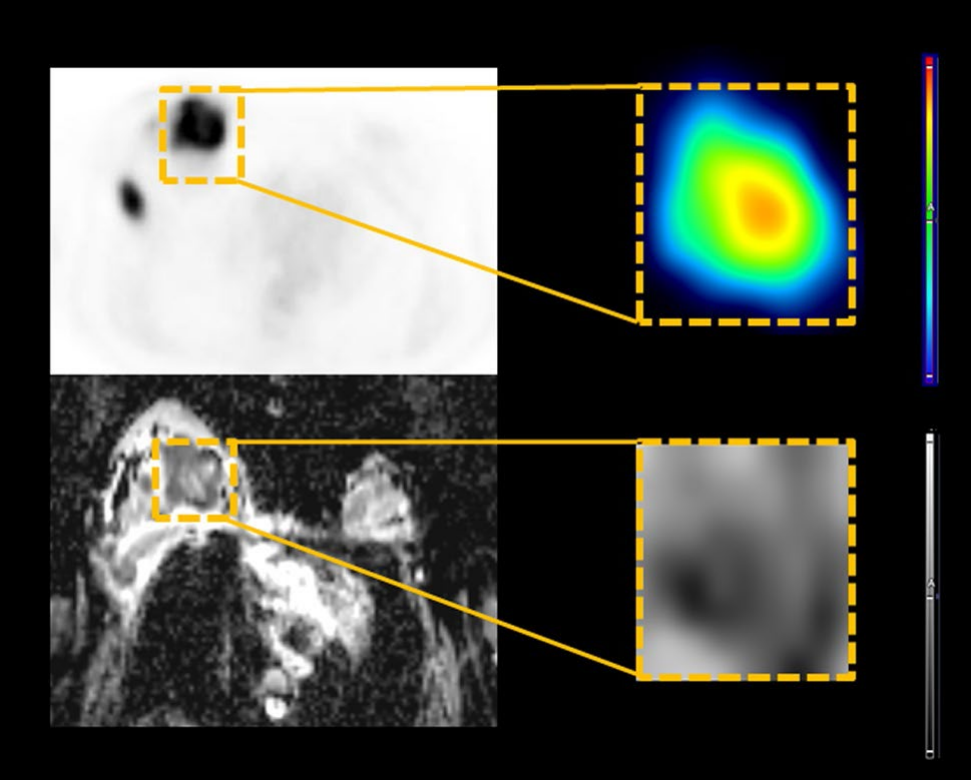
Diagram of image cropping for deep learning technique. The cubic shaped region-of-interest was selected at the largest cross-sectional area of the lesion and resized to 64 × 64 pixels. 18F-fluorodeoxyglucose (FDG) and apparent diffusion coefficient (ADC) images were obtained from positron emission tomography/computed tomography (PET/CT) and magnetic resonance imaging (MRI) scans, respectively. Baseline images were defined as PET0 and ADC0, respectively, and interim images were defined as PET1 and ADC1, respectively.

Cubic-shaped ROIs were used for image cropping for deep learning. On FDG imaging, the ROI was obtained from the largest cross-sectional area of the lesion and resized to 64 × 64 pixels. The reshape function in Tensorflow (version 1.2.1) was used for resizing. PET0 and PET1 were cropped from the baseline PET and interim PET, respectively. ADC images were aligned with the T1 images using contrast agents; the ROI was obtained from the largest cross-sectional area and was resized to 64 × 64 pixels. MRI0 images were derived from baseline ADC images, and MRI1 images were derived from the interim ADC images (Fig. 1).

The original patient data set contained a total of 56 with a 6 responder and 50 non-responder patients. Data augmentation techniques were applied to the responder patient group to prevent overfitting due to data imbalance^20,21^. The responders’ (six) images were rotated seven times in increments of 45 degrees to produce 42 images. A total of 98 patients were used for the augmented patient data set, with 48 responders and 50 non-responders.

**Figure 2:**
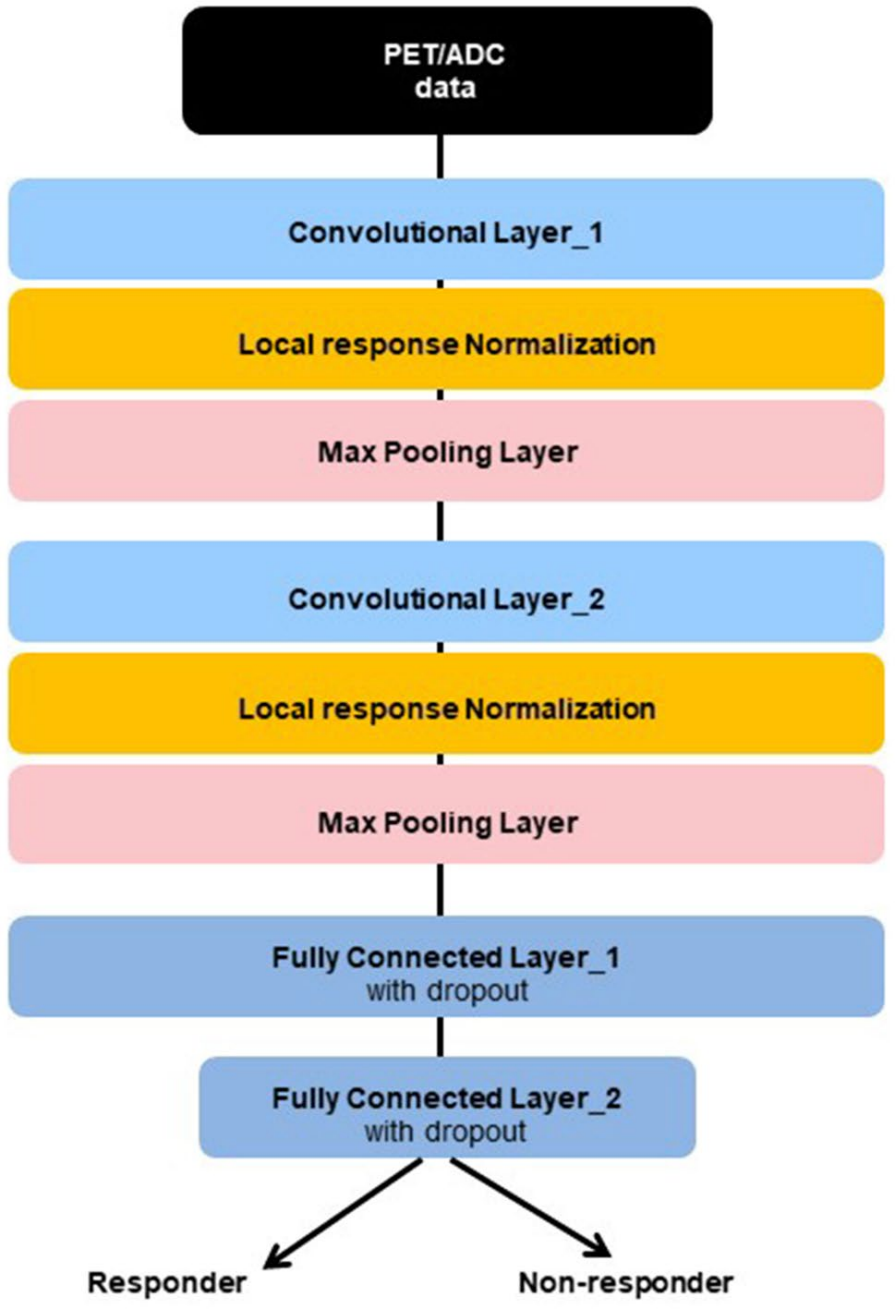
Structure of the convolutional neural network (CNN) algorithm based on Alexnet. The network used in this study contained four main layers: two convolutional layers and two fully-connected layers. The network was trained for classifying images into two types: responders and non-responders. *PET* positron emission tomography, *ADC* apparent diffusion coefficient.

The CNN structure arranges the input layers in a geometric pattern consisting of rows and columns of the image matrix^12^. It was based on Alexnet (version 2012, ImageNET large scale visual recognition challenge), using Python language (version 3.6.0), and the machine learning framework known as Tensorflow, to classify the patients into responders and non-responders. The PET/MRI image deep learning network consists of four main layers: two convolutional layers and two fully-connected layers (Fig. 2). The input layer of the CNN was used to generate convolution of a small image termed as the kernel map. The kernel map was produced in a stepwise manner by filtering of the input image. The generated kernel map included the input of the value of the extracted layer, known as the pooling layer. A 5 × 5 convolutional layer filter was adapted. A total of 32 filters were used in the first and second convolutional layers followed by a 2 × 2 filter with a max-pooling method in the pooling layer. A rectifier linear unit was used for the activation function, softmax cross-entropy was used for calculating the loss, and adaptive moment estimation (Adam) was used for loss optimization. The dropout technique was performed in the first and second fully-connected layers to prevent overfitting with the training dataset^22^.

The images were randomly assigned: 80% to the training set and 20% to the test set. The threefold validation was adapted to correct training errors and derive a more accurate estimate of predicting risk^23^. The initial training data were randomly divided into three equal subsamples. Among the three subsamples, one subsample was used as validation data for testing the model. The two residual subsamples were used as training data. The cross-validation process was repeated three times, with one repetition as the validation data for each of the three subsamples. The three results were averaged to generate a single estimate.

### Histopathological analysis

The histopathological response to chemotherapy was assessed with the Miller Payne system^24^. Grades 1–3 and grades 4 and 5 were classified as non-responders and responders, respectively.

### Statistical analysis

All statistical evaluations were performed using MedCalc software (version 16.8.4; MedCalc Software, Mariakerke, Belgium). Categorical variables were presented as numbers and percentages, and continuous variables were presented as median values with a range. Receiver operating characteristic (ROC) curve analysis was used to assess the performance of conventional imaging parameters and CNN methods for differentiating patients into responders and non-responders. Subanalysis was performed for differentiating patients into responders and non-responders in HER2-negative and triple-negative groups according to molecular subtype. Chi-squared test was applied to evaluate the association between histopathological results and molecular subtypes. The Mann–Whitney U test was used to compare the parameters before and after data augmentation. p-values of less than 0.05 were considered statistically significant.

## Results

### Patient characteristics

The patient characteristics and histologic features are described in Table 1. The median age was 49 (range 26–66) years, and the number of premenopausal women (n = 33, 59%) was slightly higher than that of postmenopausal women (n = 23, 41%). Pathological evaluation revealed that were six patients were responders (11%) and 50 were non-responders (89%). The median tumor size was 3.1 (range 2.0–8.8) cm. Stage 3 was the most common AJCC stage (n = 40, 71%) followed by stage 2 (n = 7, 13%). T2 was the most dominant T stage (n = 24, 43%), and N2 was the most dominant N stage (n = 27, 48%). 24/49 non-responders and 1/6 responders were estrogen receptor-positive. 29/49 non-responders and 3/6 responders were positive for progesterone receptors, while 20/49 non responders and 1/6 responders returned as HER2/neu-positive. The proportion of invasive ductal carcinoma was high according to the histopathological analysis (96%).

**Table 1:** Patient characteristics.

Characteristic	Value
**Age (years)**	
Median	49
Range	26–66
**Menopausal status, n (%)**	
Premenopausal	33 (59%)
Postmenopausal	23 (41%)
**AJCC stage, n (%)**	
Stage 2	12 (21%)
Stage 3	44 (79%)
**Estrogen receptor status, n (%)**	
Positive	25 (45%)
Negative	30 (53%)
No data	1 (2%)
**Progesterone receptor status, n (%)**	
Positive	32 (57%)
Negative	23 (41%)
No data	1 (2%)
**HER2/neu status, n (%)**	
Positive	21 (37%)
Negative	34 (61%)
No data	1 (2%)
**Histology, n (%)**	
Invasive ductal carcinoma	54 (96%)
Invasive lobular carcinoma	1 (2%)
Mucinous carcinoma	1 (2%)

*AJCC* American Joint Committee on Cancer, *HER2* human epidermal growth factor receptor-2.

### Prediction of treatment responses using PET and MRI parameters

**Figure 3:**
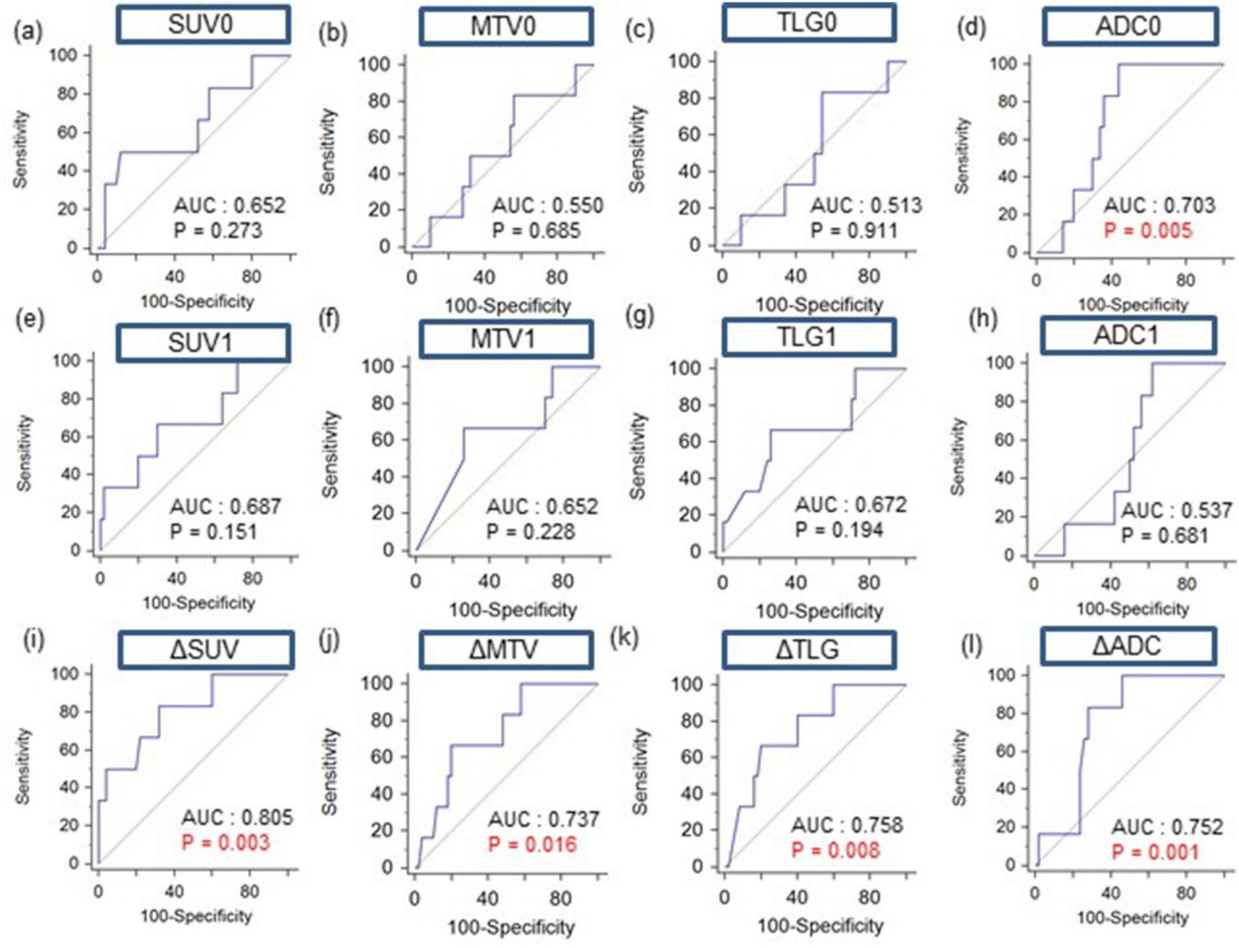
Receiver operating characteristic curve analysis for differentiating responders and non-responders of PET/CT and MRI parameters PET/CT. parameters included standardized uptake value (SUV), metabolic tumor volume (MTV), and total lesion glycolysis (TLG), and magnetic resonance imaging (MRI) parameters included mean apparent diffusion coefficients (ADC) values. Baseline values (**a**–**d**), interim values (**e**–**h**), and percentage changes in values (**i**–**l**) are depicted. *AUC* area under the curve.

ROC curve analysis for differentiating the responders from non-responders based on the PET and MRI parameters revealed that all percentage changes (ΔSUV, ΔMTV, ΔTLG, and ΔADC) were slightly higher than the baseline (SUV0, MTV0, TLG0, and ADC0) and interim values (SUV1, MTV1, TLG1, and ADC1) (Fig. 3). The AUC was the highest for ΔSUV at 0.805 (95% confidence interval (CI) 0.677–0.899; p = 0.001). The AUCs for ΔMTV, ΔTLG, and ΔADC were 0.737 (95% CI 0.602–0.845; p = 0.010), 0.758 (95% CI 0.625–0.863; p = 0.005), and 0.752 (95% CI 0.618–0.857; p = 0.001), respectively. Statistically significant differences were observed among the AUCs for these four parameters. The optimal cutoff values for ΔSUV, ΔMTV, ΔTLG, and ΔADC were − 56%, − 98%, − 99%, and 25%, respectively, with sensitivity/specificity for detecting responders of 83%/68%, 67%/80%, 67%/80%, and 83%/72%, respectively. The AUC values of interim were higher than baseline in SUV, MTV, TLG parameters, while in the ADC parameter the interim value was lower than baseline.

### Predicting responders using molecular subtype

**Figure 4:**
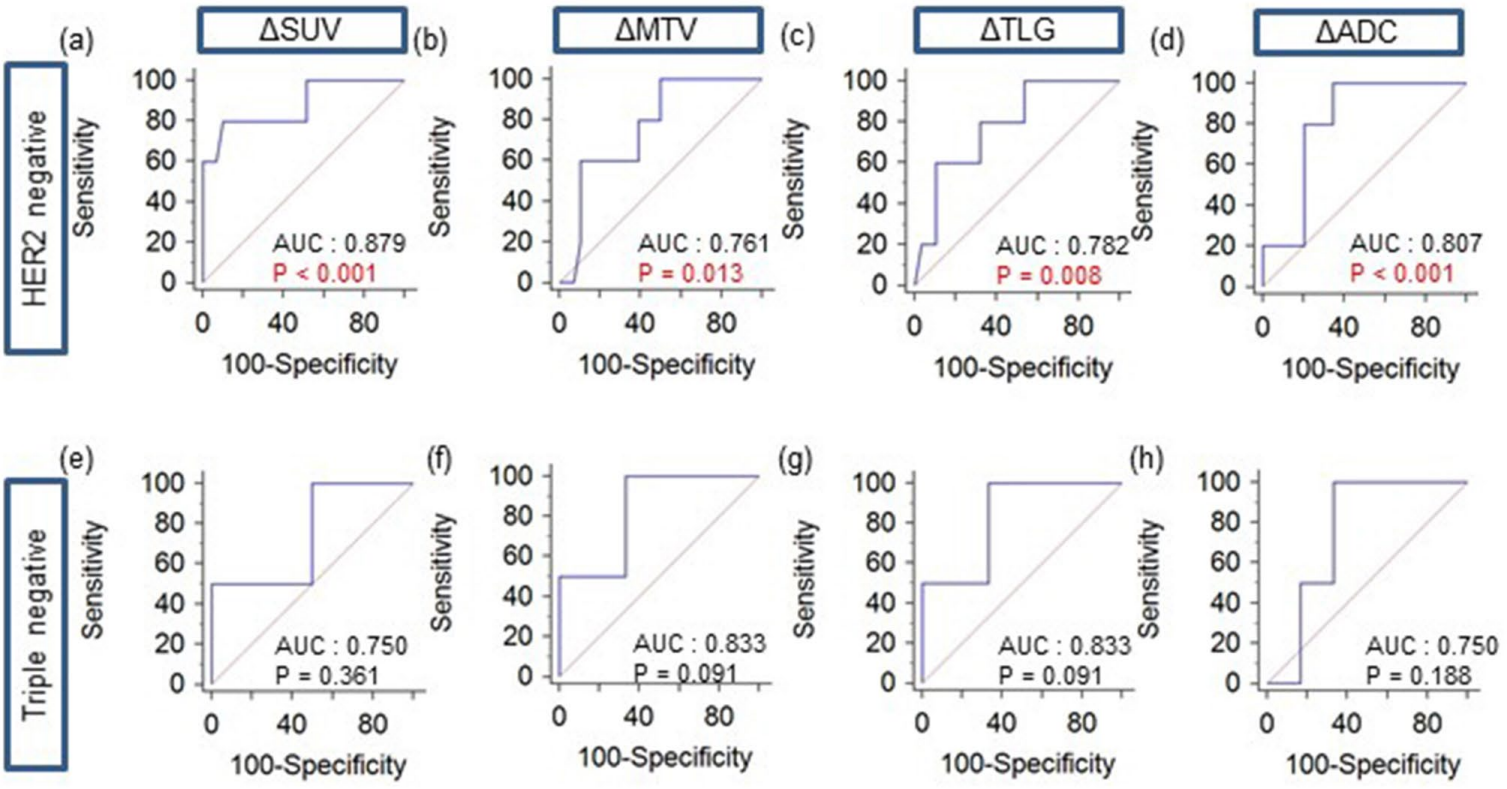
Receiver operating characteristic curves to assess changes in the standardized uptake value (ΔSUV), metabolic tumor volume (ΔMTV), total lesion glycolysis (ΔTLG), and apparent diffusion coefficient (ΔADC) for distinguishing between responders and non-responders in patients with (**a**–**d**) human epidermal growth factor receptor-2 (HER2)-negative and (**e**–**h**) triple-negative breast cancer. *AUC* area under the curve.

ROC curve analysis was used to classify responders and non-responders based on the molecular subtype with the ΔSUV, ΔMTV, ΔTLG, and ΔADC values (Fig. 4). There were five responders among 34 (15%) patients with the HER2-negative subtype (p = 0.255) and two responders among eight (25%) patients with the triple-negative subtype (p = 0.171).

In the group with the HER2-negative subtype, The AUC was the highest for ΔSUV at 0.879 (95% CI 0.722–0.965). The AUCs for ΔMTV, ΔTLG, and ΔADC were 0.761 (95% CI 0.581–0.891), 0.782 (95% CI 0.605–0.906), and 0.807 (95% CI 0.636–0.922), respectively. All values were statistically significant. The optimal cutoff values for ΔSUV, ΔMTV, ΔTLG, and ΔADC were − 61.3%, − 71.9%, − 99.3%, and 11.6%, respectively, with sensitivity/specificity for detecting responders of 80%/90%, 100%/50%, 60%/89%, and 100%/66%, respectively.

The AUC for ΔSUV was 0.750 (95% CI 0.349–0.968) for the with triple-negative subtype group, and no significant differences were noted. The optimal cutoff value was − 88.3%, with 50%/100% sensitivity/specificity for detecting responders. Both ΔMTV and ΔTLG had the highest AUC at 0.833 (95% CI 0.429–0.991); approached the borderline of significance (p = 0.091).The optimal cutoff values responders for ΔMTV and ΔTLG were − 71.9% and − 79.9%, respectively, with 100%/67% sensitivity/specificity for both parameters. The AUC for ΔADC was 0.750 (95% CI 0.349–0.968), and there were no significant differences. The optimal cutoff value was 7.8% with 100%/67% sensitivity/specificity for detecting responders.

### Comparison between the performances of conventional methods with CNN for predicting treatment responses

**Figure 5:**
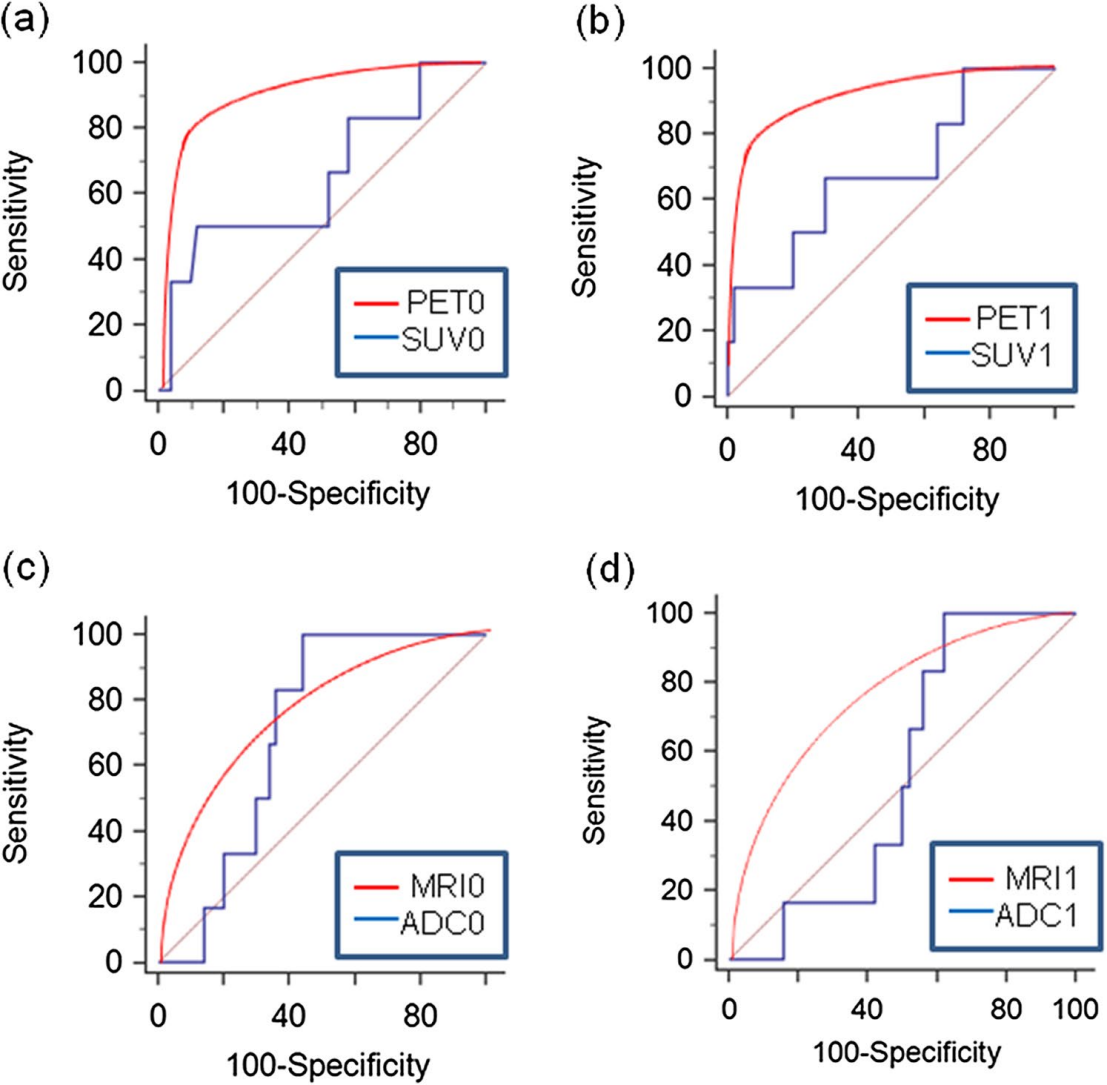
Comparisons of receiver operating characteristic curve analysis for distinguishing responders and non-responders between conventional PET/MRI parameters and convolutional neural network methods. SUV0 versus PET0 (**a**), SUV1 versus PET1 (**b**), ADC0 versus MRI0 (**c**), and ADC1 versus MRI1 (**d**). *SUV* standardized uptake value, *PET* positron emission tomography, *ADC* apparent diffusion coefficient, *MRI* magnetic resonance imaging.

As shown in Fig. 5, ROC curve analysis was used to discriminate responders and non-responders using conventional or CNN methods. The sensitivity, specificity, accuracy, and AUC values are presented in Table 2. The SUV values, which were selected as the best data from the PET data (SUV, MTV, and TLG), and ADC values were used for the conventional method. Baseline (PET0 and ADC0) and interim (PET1 and ADC1) images were used for deep learning. CNN training was conducted with 80% of the data; 20% of the test data showed the results of the responders and non-responders.

**Table 2:** Comparison between the parameters of conventional PET and MRI parameters and convolutional neural network methods for predicting pathological response to neoadjuvant chemotherapy.

	Sensitivity (%)	Specificity (%)	Accuracy (%)	AUC, median
SUV0^a^	50	88	84	0.652
PET0^b^	79	94	97	0.886
SUV1	67	70	70	0.687
PET1	72	96	95	0.980
ADC0^c^	100	56	61	0.703
MRI0^d^	18	90	85	0.602
ADC1	100	38	45	0.537
MRI1	14	90	88	0.701

*AUC* area under the curve.

^a^*SUV0* maximum standardized uptake value at baseline, *SUV1* maximum standardized uptake value on interim images.

^b^*PET0* baseline PET image data for deep learning, *PET1* interim PET image data for deep learning.

^c^*ADC0* apparent diffusion coefficient at baseline, *ADC1* apparent diffusion coefficient on interim images.

^d^*MRI0* baseline MR image data for deep learning, *MRI1* interim MR image data for deep learning, *PET* positron emission tomography, *MRI* magnetic resonance imaging.

### Performance before and after augmentation

Data augmentation was performed with the CNN values (PET0, PET1, MRI0, and MRI1) (Table 3). The threefold validation was adapted to both datasets, and the average was calculated. The reduction in accuracy was statistically significant (97% to 96%, median difference − 0.02, p = 0.046) for PET0. The sensitivity increased significantly after augmentation (79% to 100%, median difference 0.21, p = 0.046), and the specificity did not change significantly (93% to 94%, median difference 0.00, p = 0.825). The accuracy of PET1 increased in a non-significant manner (96% to 98%, median difference 0.01, p = 0.268). The sensitivity significantly increased (75% to 100%, median difference 0.25, p = 0.043), but specificity did not change significantly (96% to 95%, median difference − 0.01, p = 0.825). The accuracy, sensitivity, and specificity significantly increased for the MRI0 variables (84% to 96%, median difference 0.12, p = 0.049; 15% to 100%, median difference 0.74, p = 0.046; and 89% to 93%, median difference 0.039, p = 0.046, respectively). The accuracy (88% to 94%, median difference 0.06, p = 0.046) and sensitivity significantly increased for MRI1 (16% to 100%, median difference 0.83, p = 0.034), but specificity did not change significantly (90% to 89%, median difference − 0.01, p = 0.825).

**Table 3:** Comparisons of the areas under the curve between pre and post-augmentation values using the convolutional neural network method.

	Pre, median (range)	Post, median (range)	p value
PET0^a^	0.886 (0.834–0.951)	0.962 (0.879–0.965)	0.275
PET1	0.980 (0.966–0.983)	0.986 (0.961–0.988)	0.513
MRI0^b^	0.602 (0.555–0.622)	0.900 (0.844–0.907)	0.049
MRI1	0.701 (0.617–0.714)	0.927 (0.919–0.931)	0.049

^a^*PET0* baseline PET image data for deep learning, *PET1* interim PET image data for deep learning.

^b^*MRI0* baseline MR image data for deep learning, *MRI1* interim MR image data for deep learning, *PET* positron emission tomography, *MRI* magnetic resonance imaging.

## Discussion

The present study demonstrated the clinical impact of using CNN to predict the pathological response of NAC with PET and MRI data in patients with breast cancer. Application of the CNN method improved the accuracy of prediction. The AUC in the ROC curve analysis also improved, except for ADC0. CNN algorithms are widely used in sonography, MRI, and mammography for the detection and diagnosis of breast cancer^16^. CNN is used for the purpose of classifying data, and the well-known AlexNet, a type of CNN, shortens the computation time and improves accuracy by using two convolution layers, allowing the response of neoadjuvant chemotherapy to be well evaluated. To the best of our knowledge, no published studies have evaluated the value of CNN in predicting treatment responses to NAC among patients with breast cancer using PET and MRI. A previous study^21^ evaluated the therapeutic responses of NAC in patients with esophageal cancer using CNN methods and FDG-PET/CT and compared the results with SUVmax parameters and performed statistical analysis using texture analysis. The CNN method had the best sensitivity and specificity of all the methods. Another study assessed treatment responses in patients with bladder cancer using CNN^25^. CT images were used for pre-treatment lesion ROI on the left half of 16 × 32 pixels and post-treatment lesion ROI on the right half of 16 × 32 pixels, which were combined to produce a 32 × 32-pixel ROI. They showed sensitivity and specificity of 50% and 81% for predicting complete chemotherapy response with AUC of 0.73. This study indicates that adoption of CNN may improve the ability to distinguish between the presence or absence of a complete chemotherapy response.

Among the conventional imaging parameters, ΔSUV exhibited the best results with a sensitivity of 83% and specificity of 68% among the PET and MRI data. Similarly, a meta-analysis had shown that the SUVmax of FDG-PET/CT for predicting pathological responses in patients with breast cancer had a sensitivity of 71% and a specificity of 77%^5^. However, the study design included both post-NAC and intra-NAC values. Pahk et al.^26^ reported 86% sensitivity and 100% specificity with an intra-NAC protocol only. They focused on the luminal B molecular subtype in a relatively small cohort (n = 21), when compared to our study. Another study with an intra-NAC protocol reported an AUC of 0.78 for predicting pathological responses using relative reduction in SUVmax on PET/CT^6^. We observed a similar AUC of 0.805. The present study also measured volume-based parameters and the AUCs for ΔMTV and ΔTLG were 0.740 and 0.759, respectively. Hatt et al. reported AUCs of 0.92 and 0.91 for ΔMTV and ΔTLG, respectively, for predicting pathologic responses^27^. Despite a similar study cohort to ours, they used the scale provided by Sataloff et al. for evaluating the pathological response^28^.

The results of the ΔADC were worse than those of ΔSUV but similar to other PET parameters (ΔMTV, ΔTLG). Since the presence of natural obstacles such as membranes, cellular organs, and macromolecules interferes with the free movement of water molecules, diffusion is quantitatively measured using the ADC in biological tissues^29,30^. In the present study, the performance of ADC in evaluating pathological responses had a sensitivity of 83% and a specificity of 72%. Gao et al. performed a meta-analysis on the use of ADC for monitoring pathological responses to NAC in patients with breast cancer and reported a sensitivity of 89% and a specificity of 72%^31^. ADC values after chemotherapy showed superior predictive performance relative to ADC values before chemotherapy according to several studies^32–34^. In contrast, we observed better results before chemotherapy (ADC0). This may be due to measurement noise, which can cause low reproducibility in ADC maps^35^.

Subgroup analysis according to the molecular subtype revealed that all the changes in PET and ADC data were statistically significant in predicting the pathologic response in the HER2-negative group but not in the triple-negative group. Molecular biomarkers are correlated with patient prognosis and affect treatment planning^36^. Cheng et al. measured changes in SUV for predicting complete pathological responses in the overall and axillary lymph nodes in the HER2-negative group^37^. Groheux et al. reported that changes in SUV and TLG were best associated with complete pathologic responses in triple-negative breast cancer^38^. Koolen et al. reported that FDG uptake changes were predictive of complete pathologic responses^39^. Our study suggested that ΔMTV and ΔTLG tended to predict responders for the triple-negative molecular subtype. However, this trend was not statistically significance, probably because of the small sample size (n = 8). Further study of more samples may yield different results. The treatment responses for other molecular subtypes were not predicting owing to lack of responders among those patients.

The AUCs for predicting responders improved after augmentation. The accuracy of predicting responders improved for all parameters after augmentation, except PET0. PET0 demonstrated increased sensitivity and specificity, but the accuracy was slightly decreased. We were unable to compare the results of this model to others, as there have been no studies involving the use of a CNN to evaluate pathologic responses to NAC in patients with breast cancer. However, data augmentation contributed to parametric improvement. Thus, this approach may compensate for the imbalance in data in deep learning research.

This study had several limitations. First, our study data set was relatively small. CNNs can evaluate high-dimensional features of images, but a substantial amount of data is necessary to obtain good results^40^. K-fold validation is useful for overcoming this issue. Second, the imbalance rate was high between the responders and non-responders. Accuracy could be overestimated if the test dataset is imbalanced, and this could produce highly misleading results^20^. Third, changes between the baseline and interim images were not applied to the CNN method in contrast with the conventional method. Further research with a larger sample population is needed to address these limitations.

## Conclusion

We evaluated the pathological response of NAC for advanced breast cancer using PET/CT and MRI. The predictive performance of conventional methods was compared with that of a CNN-based model. CNNs could predict pathologic responses to NAC in patients with advanced breast cancer. CNNs have the potential to improve the diagnostic accuracy of a variety of real time clinical applications, despite their limitations. Additional studies are needed to improve the ability of this model to make clinical treatment decisions.
